# Supplementary material for: Quantitative Metabolomics to Explore the Role of Plasma Polyamines in Colorectal Cancer
Source: Int J Mol Sci. 2022 Dec 21;24(1):101. doi: 10.3390/ijms24010101 (PMC9820724; doi:10.3390/ijms24010101)

Table S1. Clinical-pathological features of patients with colorectal carcinoma

**Variables**		**Number**	**%**
**Gender**	Female	17	34.0
	Male	33	66.0
**Age**	< 65 years	19	38.0
	≥ 65 years	31	62.0
**Pathologic tumor classifcation**	1	5	10.0
	2	11	22.0
	3	28	56.0
	4	4	8.0
	NA	2	4.0
**Regional lymph nodes involvement**	0	26	52.0
	1	15	30.0
	2	7	14.0
	NA	2	4.0
**Distant metastasis**	0	41	82.0
	1	7	14.0
	NA	2	4.0
**Tumor stage**	0	1	2.0
	I	10	20.0
	II	13	26.0
	III	17	34.0
	IV	7	14.0
	NA	2	4.0
**Histologic grade**	G1	4	8.0
	G2	35	70.0
	G3	10	20.0
	NA	1	2.0
**Site**	Left	35	70.0
	Right	12	24.0
	NA	3	6.0

Figure S1. Box plots of the statistically significant inflammatory indexes values between CRC patients and healthy controls

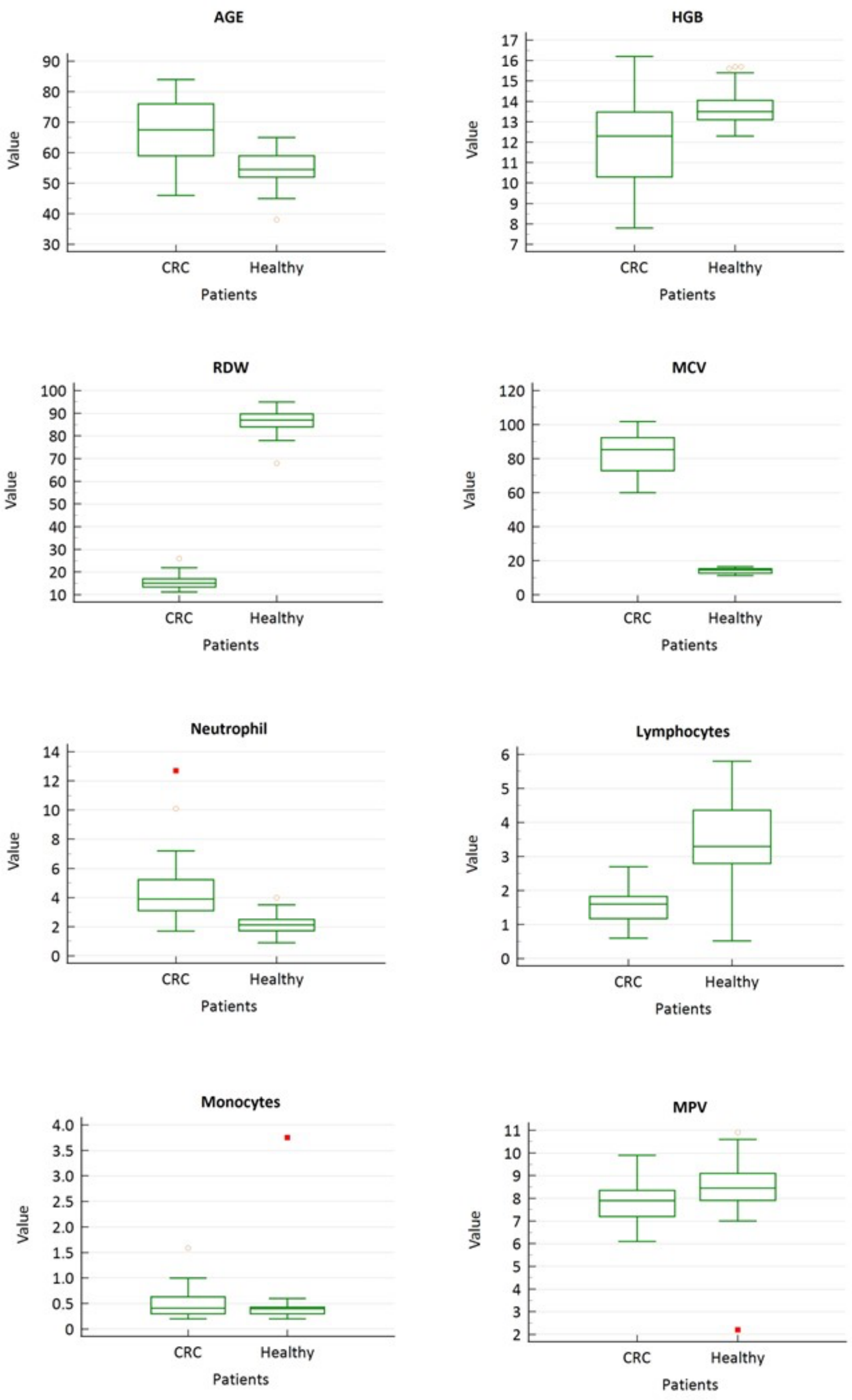

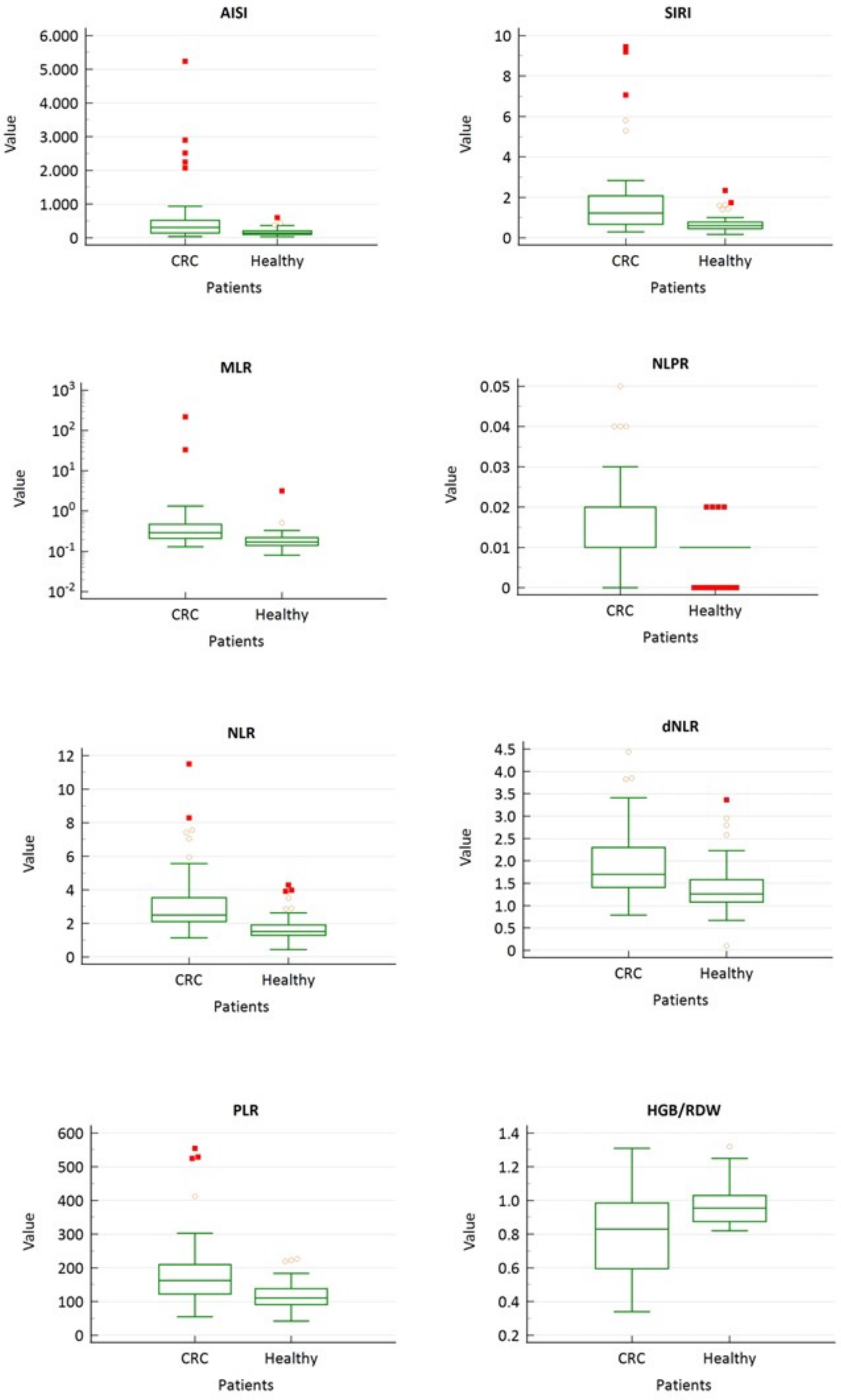
AISI
6.000
5.000
4.000
3.000
2.000
1.000
0
Value
CRC
Healthy
Patients
SIRI
10
8
6
4
2
0
Value
CRC
Healthy
Patients
MLR
10^3
10^2
10^1
10^0
10^{-1}
10^{-2}
Value
CRC
Healthy
Patients
NLPR
0.05
0.04
0.03
0.02
0.01
0
Value
CRC
Healthy
Patients
NLR
12
10
8
6
4
2
0
Value
CRC
Healthy
Patients
dNLR
4.5
4.0
3.5
3.0
2.5
2.0
1.5
1.0
0.5
0
Value
CRC
Healthy
Patients
PLR
600
500
400
300
200
100
0
Value
CRC
Healthy
Patients
HGB/RDW
1.4
1.2
1.0
0.8
0.6
0.4
0.2
Value
CRC
Healthy
Patients

Figure S2. Box plots of the statistically significant comparison between inflammatory indexes values and clinico-pathological features of CRC patients

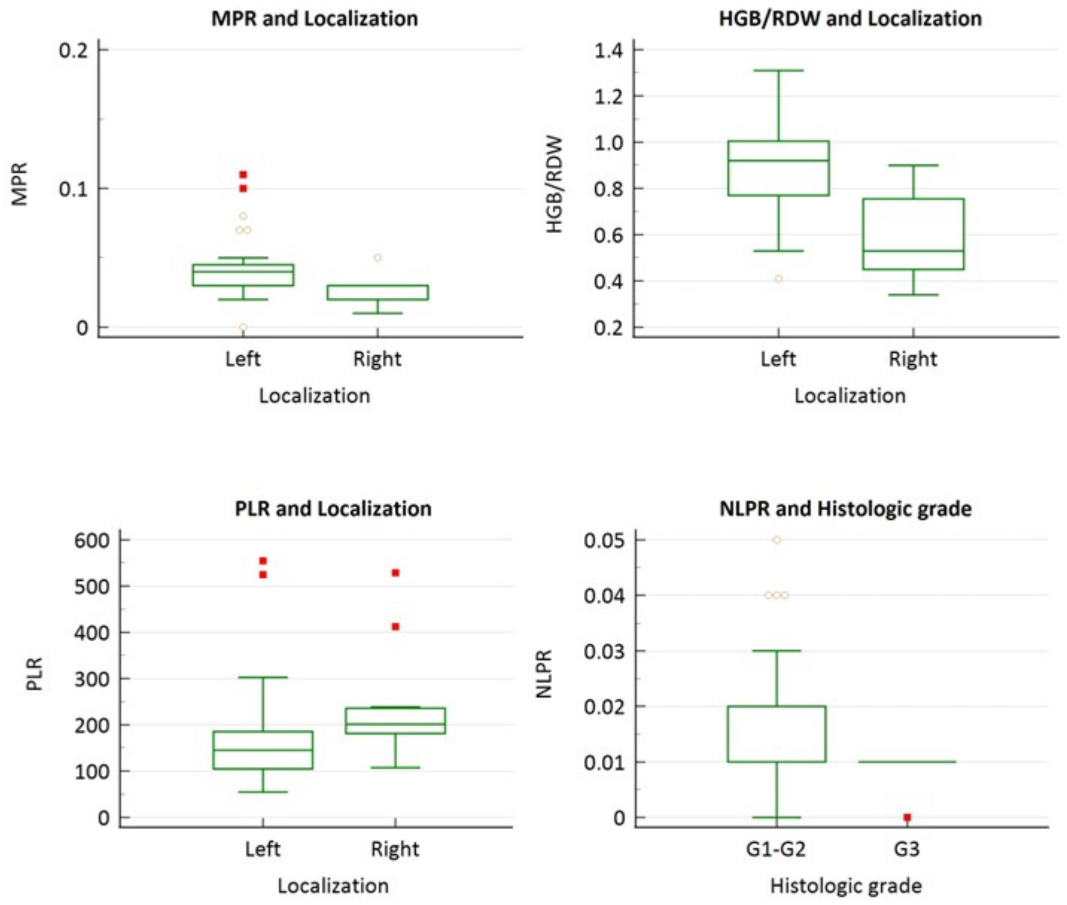

Figure S3. Box plots of the statistically significant polyamine values between CRC patients and healthy controls

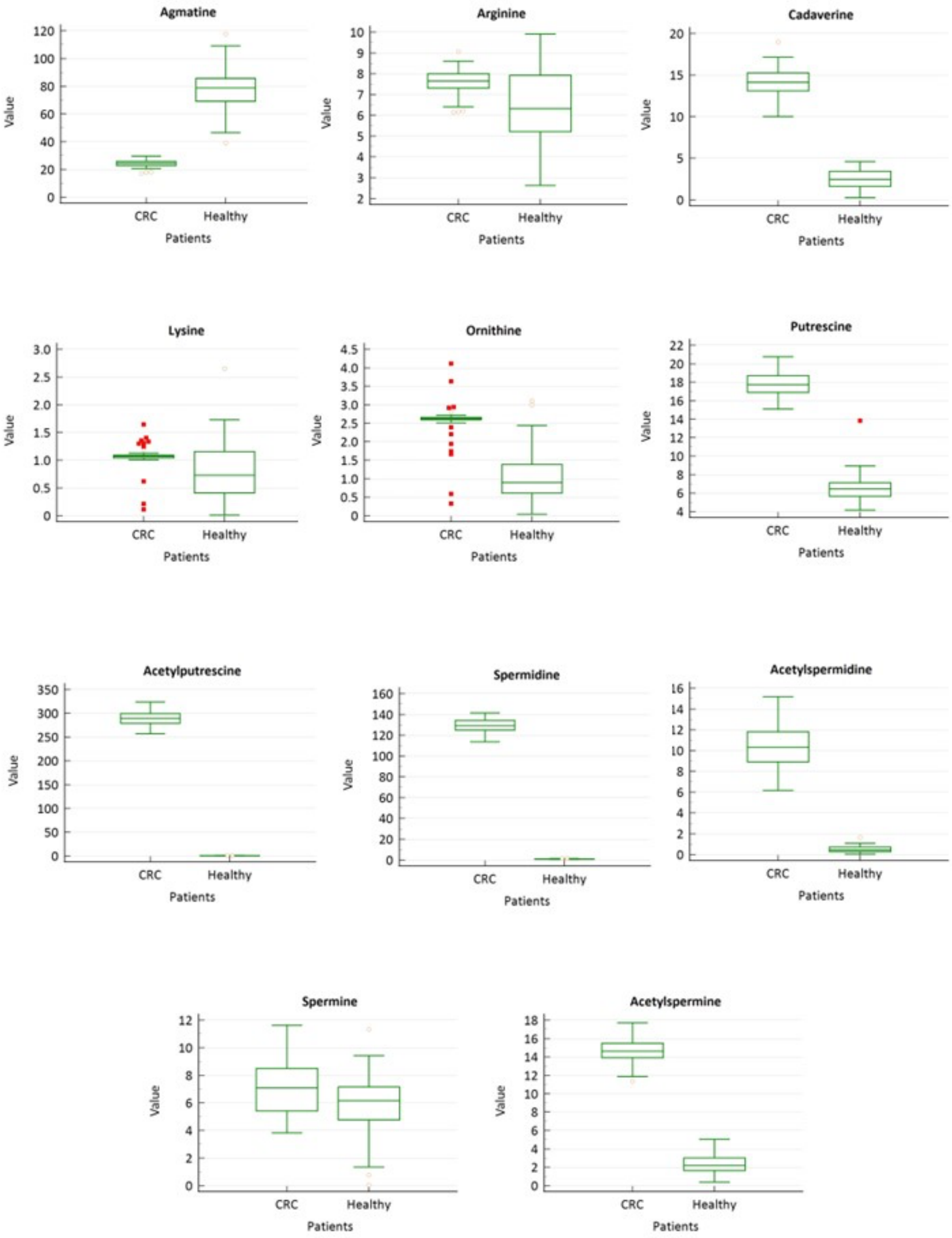

Figure S4. Box plots of the statistically significant comparison between polyamine values and clinico-pathological features of CRC patients

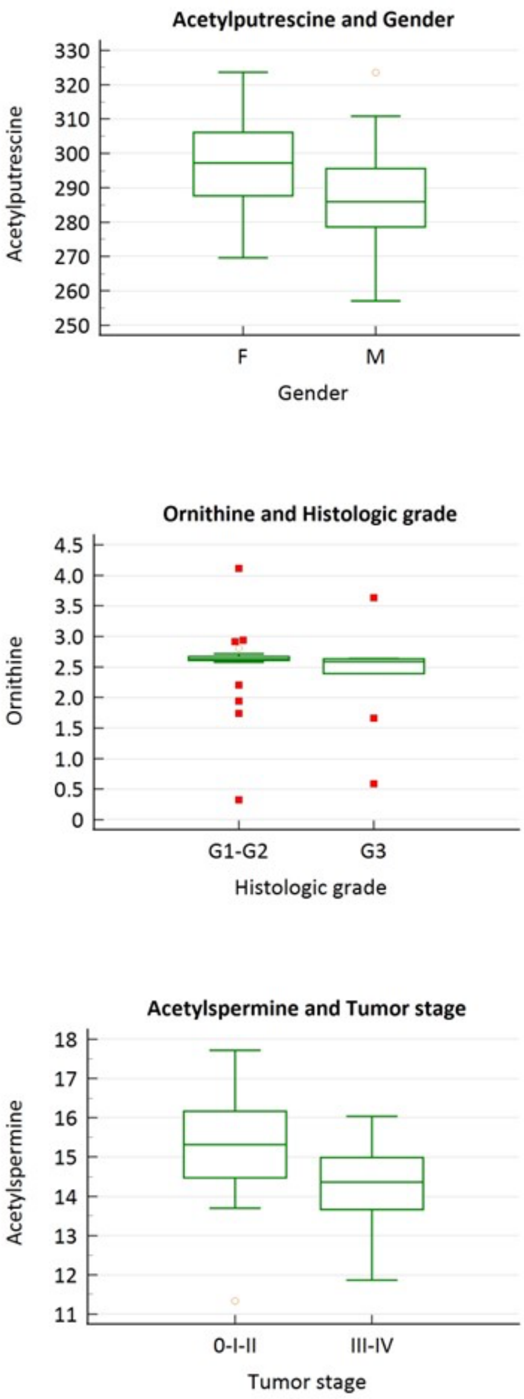

Supplement: Supplementary file 1 [file ijms-24-00101-s001.zip › ijms-2049603-supplementary.pdf]
